# Incidental detection of FGFR3 fusion via liquid biopsy leading to earlier diagnosis of urothelial carcinoma

**DOI:** 10.1038/s41698-023-00467-9

**Published:** 2023-11-18

**Authors:** Quillan Huang, Irene Mitsiades, Heidi Dowst, Neda Zarrin-Khameh, Attiya Batool Noor, Patricia Castro, Michael E. Scheurer, Guilherme Godoy, Martha P. Mims, Nicholas Mitsiades

**Affiliations:** 1 Dept. of Medicine, Baylor College of Medicine, Houston, TX 77030 USA; 2 Ben Taub General Hospital, Harris Health System, Houston, TX 77030 USA; 3 Dan L Duncan Comprehensive Cancer Center, Houston, TX 77030 USA; 4 Harvard Medical School, Boston, MA 02115 USA; 5 Boston University School of Arts and Sciences, Boston, MA 02215 USA; 6 Dept. of Pathology, Baylor College of Medicine, Houston, TX 77030 USA; 7 Dept. of Pediatrics, Baylor College of Medicine, Houston, TX 77030 USA; 8 Dept. of Urology, Baylor College of Medicine, Houston, TX 77030 USA; 9 Department of Internal Medicine, UC Davis Comprehensive Cancer Center, Sacramento, CA 95817 USA

**Keywords:** Molecular medicine, Diagnostic markers, Urological cancer

## Abstract

The rising utilization of circulating tumor DNA (ctDNA) assays in Precision Oncology may incidentally detect genetic material from secondary sources. It is important that such findings are recognized and properly leveraged for both diagnosis and monitoring of response to treatment. Here, we report a patient in whom serial cell-free DNA (cfDNA) monitoring for his known prostate adenocarcinoma uncovered the emergence of an unexpected FGFR3-TACC3 gene fusion, a BRCA1 frameshift mutation, and other molecular abnormalities. Due to the rarity of FGFR3 fusions in prostate cancer, a workup for a second primary cancer was performed, leading to the diagnosis of an otherwise-asymptomatic urothelial carcinoma (UC). Once UC-directed treatment was initiated, the presence of these genetic abnormalities in cfDNA allowed for disease monitoring and early detection of resistance, well before radiographic progression. These findings also uncovered opportunities for targeted therapies against FGFR and BRCA1. Overall, this report highlights the multifaceted utility of longitudinal ctDNA monitoring in early cancer diagnosis, disease prognostication, therapeutic target identification, monitoring of treatment response, and early detection of emergence of resistance.

## Introduction

Circulating tumor DNA (ctDNA) analysis has an established role in uncovering actionable driver mutations in solid tumors, while having potential future uses in cancer diagnosis and monitoring as well^1–3^. The concept of a “molecular response”, as defined by on-treatment reductions in ctDNA, correlates with traditional assessments such as radiographic response and survival^4–6^, but is not yet fully validated in solid malignancies. Recently, a ctDNA-guided approach to de-escalating adjuvant chemotherapy in resected stage 2 colorectal cancer resulted in reduced chemotherapy usage with no decrease in recurrence-free survival^7^. The use of ctDNA for early cancer detection is also under evaluation^8–10^. Moreover, with increasing use of ctDNA testing in clinical practice, clinicians will inevitably encounter unexpected incidental molecular findings derived from clonal hematopoiesis of indeterminate potential (CHIP) or previously undiagnosed malignancies^11^. Here we report a case where ctDNA monitoring in a patient with known prostate adenocarcinoma resulted in the expedited diagnosis of an asymptomatic urothelial carcinoma (UC) and was incorporated into the longitudinal assessment of response to treatment.

## Results

### Clinical history

A 73-year-old Hispanic male smoker with biochemically recurrent, non-metastatic castration-sensitive prostate cancer (PC), well-controlled on androgen deprivation therapy (ADT), was found on longitudinal ctDNA monitoring to have acquired somatic DNA variants that had not been reported in his PC tissue, including a FGFR3-TACC3 gene fusion, raising suspicion for a second malignancy.

Nine years earlier, the patient was diagnosed with prostate adenocarcinoma, Gleason 4 + 3, with serum prostate-specific antigen (PSA) 15.4 ng/mL. He was treated with intensity-modulated radiation therapy and 16 months of ADT with PSA nadir 0.08 ng/mL. Four years later, his PSA rose to 6.36 ng/mL; he declined salvage prostatectomy and was restarted on ADT. Since then, his serum PSA levels have remained minimally detectable and stable at <0.1 ng/mL.

**Fig. 1:**
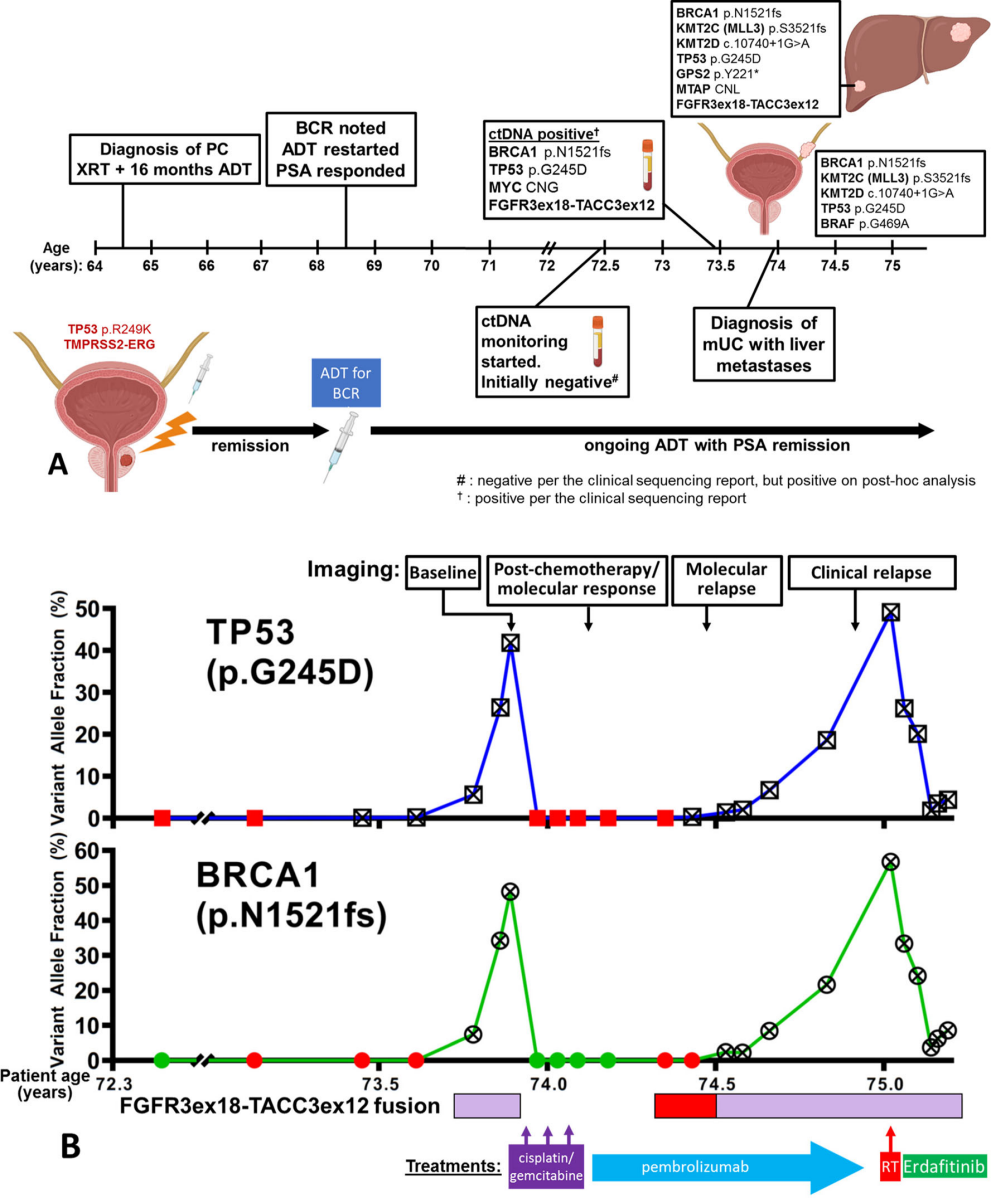
Clinical timeline and molecular studies in our patient. **A** Originally diagnosed with prostate adenocarcinoma, he was treated with radiation therapy and ADT for 16 months. Four years later, he had biochemical recurrence (BCR). He was restarted (and still is) on ADT, with serum PSA remaining minimally detectable at <0.1 ng/mL. Approximately 8 years after his PC diagnosis, NGS of the original prostate biopsy revealed a TMPRSS2-ERG rearrangement and a somatic TP53 p.R249K pathogenic LOF mutation. At that time, longitudinal ctDNA monitoring was initiated with the Tempus|xF liquid biopsy assay, yielding initially negative results per the clinical vendor’s sequencing reports. However, post-hoc analysis did demonstrate in these early liquid biopsy samples low levels of the ctDNA abnormalities that were subsequently found in the patient’s mUC (Fig. 1**B**, Supplemental Table 2). One year after initiation of ctDNA monitoring, liquid biopsies revealed multiple new (not seen previously in the NGS of the PC biopsy) ctDNA abnormalities, including TP53 p.G245D, BRCA1 p.N1521fs, MYC amplification and a fusion between FGFR3 (5’, NM_000142, exon 18) and TACC3 (3’, NM_006342, intron 11). Workup subsequently revealed a left ureteral mass and liver lesions, which were biopsy-proven to be UC and the source of the ctDNA findings. CNG: Copy number gain; CNL: Copy number loss. Created with BioRender (https://www.biorender.com/). **B** Clinical course and molecular timeline (ctDNA findings) of our patient: Samples reported as positive by the sequencing vendor in the clinical report are highlighted with a crossed symbol for the LOF mutations TP53 p.G245D and BRCA1 p.N1521fs, while the presence of the FGFR3ex18-TACC3ex12 fusion is indicated with a magenta bar (the clinical sequencing vendor does not provide VAFs for fusions). Red indicates samples that we found to be positive for the respective molecular abnormality in our own tumor-informed analysis, but had been reported as negative in real-time, because they were below the clinical vendor’s detection/reporting threshold. In our own retrospective analysis, we considered samples to be positive for each point mutation if at least 2 reads of that variant were detected in that sample. In the case of the FGFR3ex18-TACC3ex12 fusion, our threshold for positivity was 1 fusion read present. Imaging timepoints correspond to timepoints noted in Fig. 2. RT Radiation Therapy.

**Fig. 2:**
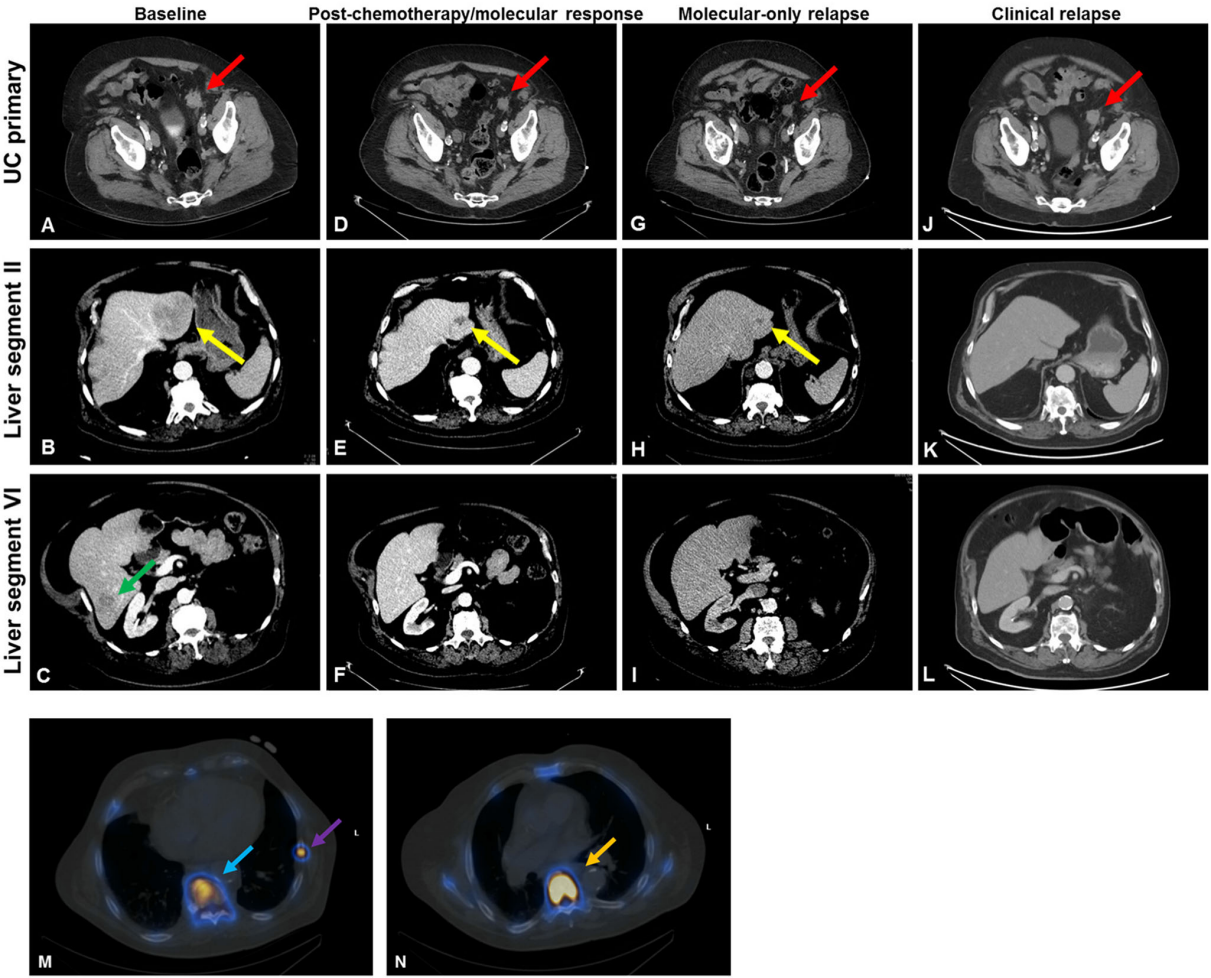
Radiographic response of mUC to treatment in our patient. **A**–**C** Pre-treatment CT demonstrated UC primary mass measuring 2.9 × 2.7 cm ((**A**) red arrow), liver segment II metastasis measuring 7.3 × 6.4 cm ((**B**) yellow arrow), and liver segment VI metastasis measuring 2.3 × 2.4 cm ((**C**) green arrow). At this timepoint, our post-hoc ctDNA analysis detected TP53 G245D at 42.9% VAF, BRCA1 p.N1521fs at 47.3% VAF, and 429 FGFR3ex18-TACC3ex12 fusion reads (normalized to a reference of 6089 total FGFR3ex18 reads). **D**–**F** CT scan after 3 cycles of cisplatin/gemcitabine chemotherapy showed minimal response in the UC primary mass measuring 2.3 × 2.1 cm ((**A**) red arrow); partial response seen in liver segment II metastasis now measuring 1.8 × 2.1 cm ((**E**) yellow arrow) and resolution of liver segment VI metastasis (**F**). At this timepoint, ctDNA abnormalities were no longer detectable by sequencing vendor’s clinical reports, although on post-hoc analysis certain abnormalities were still detectable at low VAF as detailed in Fig. 1B and Supplemental Table 2. **G**–**I** CT scan at time of molecular relapse (by sequencing vendor’s clinical reports) shows stability of UC primary measuring 2.1 × 2.1 cm ((**G**), red arrow), and ongoing radiographic response in liver segment II metastasis measuring 1.3 × 1.6 cm ((**H**) yellow arrow) and continued resolution of liver segment VI metastasis (**I**). At this timepoint, our post-hoc ctDNA analysis detected TP53 G245D at 0.26% VAF, BRCA1 p.N1521fs at 0.49% VAF, and 2 FGFR3ex18-TACC3ex12 fusion reads (normalized to a reference of 4513 total FGFR3ex18 reads). **J**–**L** CT scan at time of frank clinical relapse with progression of the UC primary mass, now measuring 2.5 × 2.4 cm ((**J**) red arrow). Liver segment II and VI metastases (**K**, **L**) are not seen. However, multifocal bone progression is better appreciated on bone scintigraphy (**M**, **N**). At this timepoint, our post-hoc ctDNA analysis detected TP53 G245D at 49.1% VAF, BRCA1 p.N1521fs at 54.7% VAF, and 1238 FGFR3ex18-TACC3ex12 fusion reads (normalized to a reference of 8890 total FGFR3ex18 reads). **M**, **N** Bone scintigraphy at time of clinical progression. M. Bone scintigraphy demonstrating a new T9 sclerotic lesion (blue arrow) demonstrating Tc-99m MDP uptake consistent with a bony metastasis. Biopsy subsequently demonstrated metastatic UC (Fig. 3). Also shown is an additional area of Tc-99m MDP uptake at the left ninth rib (purple arrow), also consistent with bony metastasis. **N** Additional area of Tc-99m MDP uptake at T7 (orange arrow), consistent with bony metastasis. In addition to T7, T9, and left ninth rib lesions shown above, bone scan also demonstrated abnormal radiotracer uptake suspicious for metastatic disease at T3, T8, T10, right orbit, sternum, right second rib, and proximal femurs (not shown).

**Fig. 3:**
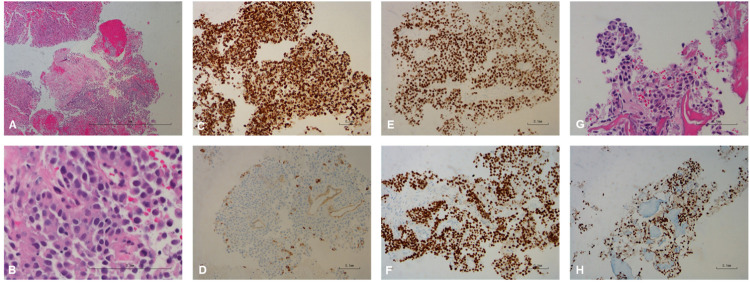
Pathology of liver segment II biopsy and bone (spine) T9 metastases. **A**, **B** Biopsy of liver lesion at time of UC diagnosis, showing reactive hepatocytes (upper left) and metastatic UC (lower half), with hemorrhage and necrosis (lower left). Hematoxylin & Eosin stain, low power. Scale bar represents 1 mm (**A**). In higher magnification: Malignant cells have focal papillary features (top), with abundant eosinophilic cytoplasm, round homogenous nuclei and small nucleoli. Hematoxylin & Eosin stain, high power. Scale bar represents 0.1 mm (**B**). **C**–**F** The tumor cells stain positive for CK7 (**C**), focal positive for CK20 (**D**), positive for P63 (**E**), and positive for GATA3 (**F**), consistent with UC. Scale bars represent 0.1 mm. **G**, **H** Biopsy of T9 bone lesion at time of clinical progression showing metastatic UC. Hematoxylin & Eosin stain, medium power (**G**). Tumor cells stain positive for GATA3 (**H**). Scale bars represent 0.1 mm.

**Fig. 4:**
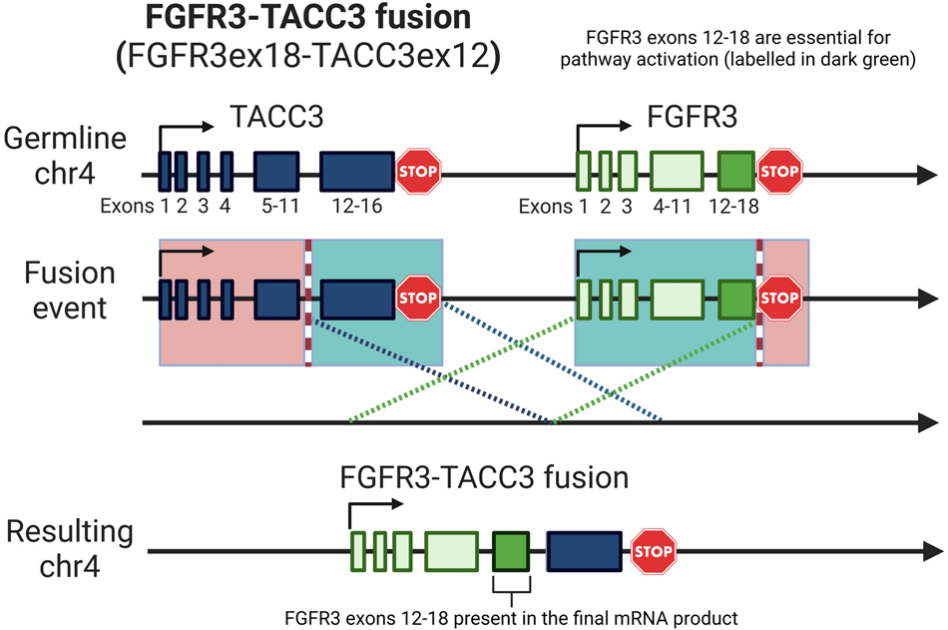
Schematic representation of the FGFR3ex18-TACC3ex12 fusion detected initially by ctDNA and then confirmed in the liver metastasis in our patient. The translocation juxtaposes exons 1-18 of FGFR3 to exon 12 of TACC3, and is a commonly-reported fusion product seen in urothelial and other carcinomas^73^. FGFR3 exons 12-18 encode for the kinase domain, and are critical for enzymatic activity of the fusion product. The coiled coil domain of TACC3 promotes dimerization of the fusion protein, which results in autophosphorylation of its kinase domain and constitutive kinase activity. Created with BioRender (https://www.biorender.com/).

Approximately 8 years after his initial presentation, the patient underwent next-generation sequencing (NGS) of his original PC biopsy tissue (Fig. 1A) and was started on longitudinal cell-free DNA (cfDNA) monitoring using the commercially available Tempus platform as part of a Precision Oncology initiative at our institution. Liquid biopsies for ctDNA monitoring were performed at the time of each follow-up visit, on average three months apart. Initially these reported no abnormalities, but approximately nine years from his initial PC presentation, the clinical reports of the liquid biopsies began to demonstrate multiple molecular abnormalities at rising variant allele fractions (VAFs), including TP53 p.G245D, BRCA1 p.N1521fs, MYC amplification and FGFR3ex18-TACC3ex12 fusion (Figs. 1A, B). Because none of these were present in the patient’s original PC tissue, and as FGFR3-TACC3 fusions are very rare in PC (Supplemental Table 1), we pursued workup for a second malignancy^12^. Imaging showed a left ureteral mass (with biopsy revealing high-grade UC, positive for GATA-3, P63, CK7, and CK20), a pathologic para-aortic node, and liver lesions in segments 2 and 6 (Figs. 1A, 2A–C, 3). Liver biopsy confirmed metastatic UC (mUC) (Fig. 3). NGS performed on the liver biopsy specimen demonstrated the FGFR3ex18-TACC3ex12 fusion, which, interestingly, was not detected in the primary UC biopsy (Figs. 1A, 4).

Systemic treatment for mUC was initiated with cisplatin and gemcitabine. After one cycle, all molecular abnormalities disappeared from the patient’s liquid biopsy clinical report (Fig. 1B). After three cycles of chemotherapy (out of six planned), there was concern for cisplatin nephrotoxicity. Repeat imaging showed response at all sites of disease (Fig. 2D–F). Given radiographic as well as molecular response with elimination of detectable ctDNA findings from the clinical NGS reports, it was decided to de-escalate treatment and switch to immunotherapy with pembrolizumab. Six months later, the clinical reports of cfDNA NGS began again to show abnormalities seen previously in the patient’s UC, including TP53 p.G245D as well as, eventually, the BRCA1 p.N1521fs and the FGFR3ex18-TACC3ex12 fusion (Fig. 1B). Restaging scans at that time did not show radiographic progression of disease (Fig. 2G–I).

Approximately four months after the reappearance of the UC-associated ctDNA abnormalities, restaging imaging showed a questionable new sclerotic lesion within the T9 vertebral body. All prior sites of disease, including the urothelial primary site and liver metastases, were stable in size. After discussion with Radiology, a decision was made to obtain a bone scan, which was performed one month later and was convincing for new bony metastatic disease, with sclerotic lesions showing radiotracer uptake at T7-T10, T3, sternum, right second rib, and left ninth rib (Fig. 2M, N). Repeat body imaging also demonstrated minimal enlargement of the UC primary, while the known liver metastases remained stable (Fig. 2J–L). During this time, the VAFs of the UC-associated molecular abnormalities in the cfDNA continued to rise **(**Fig. 1B), and, for the first time, additional gain-of-function (GOF) variants FGFR2 p.F276C and NRAS p.Q61L were reported in the clinical results by the NGS vendor. Meanwhile, the patient’s PSA remained minimally detectable at <0.1 ng/mL, and no PC-related mutations were found in the cfDNA. Thus, clinical suspicion was much higher for progressing UC rather than PC relapse. At this time, the patient was offered a therapy switch for presumed progressing UC, but ultimately it was decided to first pursue a confirmatory biopsy of the T9 bone lesion, which showed metastatic UC (Fig. 3). Unfortunately, this tissue was inadequate for NGS testing. Radiation treatment was administered to the T9 bone lesion, with a rapid reduction in ctDNA VAFs of UC-associated mutations (Fig. 1B, Supplemental Table 2). The patient was then initiated on systemic therapy with the FGFR inhibitor erdafitinib, which initially resulted in further improvement of the ctDNA VAFs of the UC-associated mutations. However, very quickly, the VAFs for TP53 p.G245D and BRCA1 p.N1521fs began to rise again (Fig. 1B, Supplemental Table 2), while the ctDNA presence of FGFR3ex18-TACC3ex12 and FGFR2 p.F276C declined dramatically (Supplemental Tables 2 and 3). At the same time, the NRAS p.Q61L VAF increased rapidly (Supplemental Table 2). Collectively, these observations suggest that FGFR3ex18-TACC3ex12 and FGFR2 p.F276C are sensitive to erdafitinib, while NRAS p.Q61L can serve as a mechanism of resistance to it.

### Retrospective re-analysis of cfDNA sequencing data

Throughout the patient’s course detailed above, clinical-grade NGS results were provided by the clinical sequencing vendor to the treating physicians in real time. While NGS on the cfDNA platform is performed at 5,000x coverage, clinical reports are limited by reporting cutoffs set by the vendor, including VAF of 0.1% for missense variants and 0.5% for insertions/deletions (indels), as well as lack of reporting VAFs for fusion products. We, therefore, retrospectively sought to better understand our patient’s disease course by re-aligning the raw sequencing data, as described in the Methods section, for possible UC-derived variants that were present below the vendor’s reporting thresholds.

In cfDNA samples from the period preceding the diagnosis of mUC, our own tumor-informed re-alignment and re-analysis found the TP53 G245D and BRCA1 N1521fs mutations to be present 12 (at least) and 8 months, respectively, prior to their earliest appearance in the clinical reports, at VAFs of 0.24% and 0.04%, respectively. FGFR3ec18-TACC3ex12 was not detected at any new earlier time point compared to the clinical reports (Fig. 1B and Supplemental Table 2).

After initiation of cytotoxic chemotherapy for mUC, the clinical NGS reports were negative for any UC-associated cfDNA findings for approximately six months. However, our own tumor-informed re-alignment and re-analysis found the TP53 G245D to be detectable in the cfDNA throughout this entire period, while we detected the BRCA1 N1521fs in cfDNA samples collected 2 months prior to its re-appearance in the vendor’s clinical reports (Fig. 1B). Details on all analyzed variants are presented in Supplemental Table 2.

Collectively, our own tumor-informed re-alignment and re-analysis shows that UC-associated somatic mutations could be detected in the cfDNA, albeit at low VAFs, at least 12 months prior to their earliest appearance on the vendor’s clinical reports; at least 18 months prior to clinical UC diagnosis; and at least 12 months prior to definitive radiographic progression after first-line therapy.

### Supplemental tissue genomic testing

The clinical sequencing vendor provides a homologous recombination deficiency (HRD) test, in which a score computed based on a proprietary algorithm on formalin-fixed tissue samples is designed to predict the probability that a tumor’s gene expression profile correlates with benchmarks of a HRD phenotype, such as those found in tumors with biallelic BRCA loss^13^. Testing of both the UC primary and the liver metastasis yielded a highly positive score of 88/100 and 96/100 by this assay, respectively (assay cutoff: >50/100 is considered HRD+).

## Discussion

**Fig. 5:**
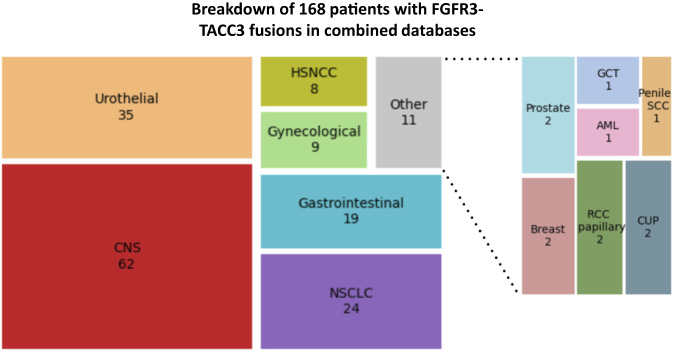
Treemap of FGFR3-TACC3 fusion-positive cancers reported across a series of public databases. AACR GENIE cohort v12.0, cbioportal (MSK-IMPACT, TCGA PanCancer Atlas, UMich Metastatic Solid Cancers, Broad/Dana-Farber MSS Mixed Solid Tumors, China Pan-cancer), and internal unpublished data from Baylor College of Medicine^19–26^. Combined, the three databases (accessed 9/22/22) contained genetic sequencing data from 213,438 unique patients, of whom 168 had tumors containing FGFR3-TACC3 fusions, for an overall frequency of 0.079%. The breakdown of tumor types of the 168 patients with FGFR3-TACC3 fusions is shown. The highest number of cases of this fusion were reported in central nervous system cancers, followed by urothelial cancers. For more details, see Supplemental Table 1. Created with Squarify (https://github.com/laserson/squarify).

Our patient’s mUC was detected, while asymptomatic, by liquid biopsy cfDNA NGS which uncovered molecular alterations not previously seen in his PC. While some of these newly detected mutations (e.g. TP53 and BRCA1) are relatively tissue-nonspecific and could potentially be acquired during clonal evolution of his PC, the FGFR3-TACC3 fusion is extremely rare in PC (Fig. 5 and Supplemental Table 1) and was highly suggestive of a new UC primary.

Fibroblast growth factor receptors (FGFRs) play important roles in cellular growth, differentiation, and survival^14^. FGFR aberrations can include gene amplification, GOF point mutations, and fusions, leading to constitutive activation of downstream signaling pathways promoting carcinogenesis^15^. FGFR fusions most commonly involve FGFR2/3 and can produce functional fusion proteins with constitutively active tyrosine kinase domains (Fig. 4 and^16^). While numerous fusion partners have been described for both FGFR2/3, one of the most common fusion partners with FGFR3 across multiple tumor types is transforming acidic coiled-coil-containing protein 3 (TACC3). This fusion likely occurs via a tandem duplication event on chromosome 4^15–17^. In addition to altered FGFR3 kinase signaling, these fusions may also promote carcinogenesis via mitotic defects^18^. While FGFR alterations have been documented in virtually all types of solid cancers, the FGFR3-TACC3 fusion is extremely rare in PCs, which raised suspicion of a second primary malignancy in our patient^12,19–26^ (Fig. 5 and Supplemental Table 1).

FGFR3 fusions are seen in approximately 5% of UCs^27–29^. The FGFR3ex18-TACC3ex12 fusion was present in our patient’s cfDNA and liver metastasis, but not in his primary UC (Fig. 1A and Supplemental Table 2). Furthermore, the FGFR3ex18-TACC3ex12 fusion frequency in the liver metastasis at the time of diagnosis was consistent with it being only a subclonal event: 65 fusion reads/1540 total reads at FGFR3ex18, yielding an allele frequency of 4.2%, vs. TP53 p.G245D VAF of 74.2% and BRCA1 N1521fs VAF of 68.6%. Although FGFR3 point mutations are reported to be an early event in UC pathogenesis and are seen more frequently in non-muscle-invasive UC, the same is not necessarily true of FGFR3 fusions^30^. Subsequent ctDNA reads of the FGFR3ex18-TACC3ex12 fusion, normalized to total FGFR3ex18 reads and to the TP53 mutation VAFs, were elevated at the time of clinical progression compared to both the original liver metastasis and the pre-treatment cfDNA, consistent with clonal expansion of the fusion-harboring clone (Supplemental Table 2 and 3). The co-existence of BRCA1 and TP53 loss-of-function (LOF) mutations, including in the primary UC tumor, also suggest that our patient’s tumor may have harbored a tandem duplicator phenotype that may have produced the FGFR3ex18-TACC3ex12 fusion in a subclonal population^15,16^. This phenotype has been best described in breast and ovarian cancers, and combined abrogation of BRCA1 and TP53 is described to result in genome-wide instability that manifests as increased tandem duplications.

Erdafitinib is a FGFR1-4 inhibitor which has received FDA accelerated approval for mUC harboring susceptible FGFR alterations^31^. The approval was based on a phase 2 single arm study enrolling UC patients with either FGFR3 mutations or FGFR2/3 fusions. The response rate to erdafitinib was higher in patients with FGFR mutations (49%) than in patients with FGFR2/3 fusions (16%). The most common fusion was *FGFR3:TACC3v1*; four out of eleven (36%) of these patients had objective responses. A confirmatory phase 3 study (THOR) comparing erdafitinib to chemotherapy (investigator’s choice) in patients with FGFR2/3 alterations has been presented in abstract form, with results showing a significant overall survival benefit for erdafitinib^32^. In our patient, erdafitinib had an initial suppressive effect on the TP53 p.G245D and BRCA1 p.N1521fs VAFs, but they quickly began to rise again (Fig. 1B, Supplemental Table 2), while the cfDNA presence of FGFR3ex18-TACC3ex12 and FGFR2 p.F276C declined dramatically (Supplemental Table 3). Our longitudinal cfDNA monitoring suggests that the FGFR3ex18-TACC3ex12 and FGFR2 p.F276C kinases are sensitive to erdafitinib, while NRAS p.Q61L can serve as a mechanism of resistance to it.

In mUC, the optimal number of cycles of platinum-based chemotherapy is not known; although 6 cycles is a traditional goal, 3–5 cycles may be noninferior^33^. There are no guidelines for therapy de-escalation based on clinical factors, although, in practice, toxicity often is dose-limiting with an unknown impact on effectiveness. In early-stage solid tumors, ctDNA levels after treatment correlate with minimal residual disease and predict for relapse^34–36^. De-escalation of chemotherapy via utilizing cfDNA monitoring is a strategy under investigation in multiple cancer types^7^. In the neoadjuvant setting, clearance of ctDNA after neoadjuvant therapy correlates with pathologic complete response^37,38^. Our patient’s rapid initial decline in tumor-associated variants was reassuring for a good clinical response to chemotherapy and contributed to the decision to transition to maintenance immunotherapy. Although our patient ultimately had a molecular relapse followed by a clinical relapse, his clinical progression-free interval exceeded the median reported in trials of maintenance immunotherapy in mUC^39^.

Our patient’s UC carries several molecular abnormalities which can be followed longitudinally by ctDNA analysis as a complement to conventional imaging. After one cycle of chemotherapy, these molecular abnormalities dropped below clinical reporting threshold. Reappearance of known tumor-associated variants, as in our patient, serves as an early warning sign that may precede impending clinical relapse. Moreover, appearance of new variants can suggest clonal evolution and emergence of resistance mechanisms^40^, such as, in this case, NRAS p.Q61L. On-treatment longitudinal cfDNA monitoring correlates well with time-to-treatment failure and progressive disease, and should prompt close disease monitoring^6,41,42^. There is emerging evidence that intervening early on the basis of ctDNA changes alone can delay time to frank clinical progression in estrogen receptor-positive breast cancer^43^. However, more validation is needed before basing management decisions on ctDNA alone can be considered standard-of-care in any tumor type.

Our patient’s UC also harbored a BRCA1 LOF mutation detectable in both blood and tissue (the latter with a VAF of 88.3%, suggestive of biallelic loss). Consistent with this, his HRD-RNA score (an assay validated to detect transcriptional signatures of HRD)^13^, indicated HRD-high phenotype in both his primary UC site and his liver metastasis. LOF mutations in BRCA1/2 are present in 5–10% of patients with UC, although only a minority of them are predicted to have a HRD phenotype, probably because the rest lack locus-specific loss of heterozygosity^21,44,45^. By the commercially available RNA-based HRD test, 3.4% of bladder cancers are homologous recombination-deficient^13^. Defects in homologous recombination can predict sensitivity to platinum-based chemotherapy in urothelial cancers^44,46,47^. After platinum-based chemotherapy, the poly (ADP-ribose) polymerase (PARP) inhibitor rucaparib extends PFS in mUC patients with biomarkers of HRD or HRD-associated mutations^48^. Responses to olaparib have also been reported in patients with BRCA1 LOF mutations^49^. Untreated, platinum ineligible UC patients harboring homologous recombination repair gene mutations including BRCA1/2 may also benefit from the PARP inhibitor olaparib in combination with the PD-L1 inhibitor durvalumab^50^. Our patient’s BRCA1 mutation VAFs suggest that this molecular event was acquired relatively early in the evolution of this tumor, which is also supported by the high HRD scores at both primary site and metastasis. Therefore, although no PARP inhibitor is currently FDA-approved for UC in any line of therapy, PARP inhibition remains a consideration for a later line of therapy if standard-of-care options have been exhausted and if genetic testing at that time continues to show a dominant BRCA1-mutated/HRD+ clone.

Our patient’s primary UC tumor harbored a subclonal BRAF p.G469A mutation, which was not detected within the liver metastasis. This mutation was never reported in any ctDNA clinical reports. However, our own re-alignment of the raw sequencing files revealed a positive sample, which was collected near the time of initial diagnosis (i.e., at peak disease burden), with a VAF of 0.04%, and this mutation again became detectable at a low VAF of 0.02–0.03% intermittently in samples collected at the time of clinical progression (again, a timepoint of high tumor burden). Oncogenic BRAF mutations have been classified by their mechanism of action^51,52^. Class I BRAF mutations include classical BRAF p.V600E mutations, where BRAF signals as an active monomer in a RAS-independent fashion. The combination of the BRAF inhibitor dabrafenib and the MEK inhibitor trametinib carry a tumor-agnostic FDA indication for patients with tumors harboring a BRAF V600E mutation based on two basket trials^53,54^. Class II BRAF mutations, including the p.G469A that was seen in our patient, signal as activated heterodimers and are resistant to existing BRAF inhibitors such as dabrafenib, but have been reported to have some clinical susceptibility to off-label MEK inhibition in limited case reports^55,56^. In our patient, the subclonal nature of the BRAF mutation, its complete absence from the metastatic site, as well as the lack of strong evidence for activity of BRAF or MEK inhibition against non-V600 BRAF mutants, make BRAF-targeted therapy less attractive.

While our patient’s UC was detected incidentally as part of a cfDNA surveillance program intended to monitor his known PC, this case also illustrates the potential of cfDNA for early cancer detection, especially for histologies without screening guidelines (such as UC). The PATHFINDER study evaluated a multi-cancer early detection (MCED) methylation-based screen in participants without a known cancer diagnosis, detecting a cancer signal in 1.4% of patients, of whom 38% were ultimately diagnosed with cancer^9,10^. In the parallel SYMPLIFY study of participants presenting with non-specific symptoms felt to be potentially concerning for cancer, the MCED assay demonstrated a positive predictive value of 75.5%, although sensitivity towards detecting cancer was lower in stage I vs stage IV patients (24.2% versus 95.3%)^57^. The CancerSEEK assay, a PCR-based assay directed against 16 cancer-associated genes, demonstrated sensitivity of 15.6% when backed by PET imaging^58^. Use of cfDNA for multiple cancer screening continues to be an area of active investigation^8^.

In conclusion, our case report highlights the ability of longitudinal cfDNA monitoring to transform clinical Oncology practice through early cancer detection, identification of personalized therapeutic targets, monitoring response to therapy, predicting clinical relapse/progression and characterizing the mechanism of drug resistance.

## Methods

### Molecular testing

All NGS testing was performed using the commercially available platforms by Tempus, Inc (Chicago, IL). Both the initial sequencing of the patient’s PC tissue as well as the sequencing of his UC biopsy samples were performed with the Tempus|xT assay, which is a 648-gene NGS-based test with depth of coverage of 500x. Longitudinal cfDNA monitoring was performed using the Tempus|xF assay, which is a 105-gene NGS-based test with depth of coverage of 5000x^59^. Functional assessment of homologous recombination deficiency (HRD) was determined by the Tempus HRD-RNA algorithm^13^ in the patient’s tissue samples. This algorithm uses gene expression data from 16,750 RNA-seq samples to predict the probability that a tumor’s gene expression profile (assessed by evaluating RNA expression of 20,000 genes) correlates with well-characterized benchmarks of the HRD phenotype^13^.

### Data analysis

The patient was enrolled in a specimen acquisition research protocol (approved by the Baylor College of Medicine IRB and administered by the Integrated Biobanking Shared Resource at the Dan L Duncan Comprehensive Cancer Center) and provided written informed consent. Re-alignment of raw sequencing data was performed post-hoc (not available for real-time clinical decision-making) by our team (I.M.). The raw NGS data, provided by the sequencing vendor in the form of FastQ files, were trimmed and aligned to UCSC Golden Path’s hg19 (GRCh37) 2009 version. The resulting BAM files were then sorted and run through the MarkDuplicates program from the package Picard to remove PCR duplicate reads. The SVIM package was used to identify structural variants. In our own analysis, we considered samples positive for each point mutation if at least 2 reads of that variant were detected, and we considered samples positive for the FGFR3ex18-TACC3ex12 fusion if at least 1 fusion read was present. The squarify package was used to generate the treemap in Fig. 5.

### Reporting summary

Further information on research design is available in the Nature Research Reporting Summary linked to this article.

### Supplementary information

Supplemental File

REPORTING SUMMARY

## Data Availability

The datasets used and/or analyzed during this study are available in the SRA repository at https://www.ncbi.nlm.nih.gov/sra/PRJNA988144.

## Code availability

All code used in this analysis, including the DNA pipeline and post-processing analyses, is available at https://github.com/ringenomics/FGFR3fusion^60–72^.
