# Supplementary material for: Incidental detection of FGFR3 fusion via liquid biopsy leading to earlier diagnosis of urothelial carcinoma
Source: NPJ Precis Oncol. 2023 Nov 18;7:123. doi: 10.1038/s41698-023-00467-9 (PMC10657397; doi:10.1038/s41698-023-00467-9)
Supplement: Supplementary file 1 — Supplemental File [file 41698_2023_467_MOESM1_ESM.pdf]

Supplemental Table 1

Tumor type	GENIE	cbioportal	Baylor	Total
CNS	43	11	8	62
urothelial	20	9	6	35
NSCLC	12	11	1	24
gastrointestinal	1	17	1	19
gynecologic	3	5	1	9
HNSCC	2	4	2	8
breast	2	0	0	2
prostate	1	0	1	2
RCC, papillary	0	2	0	2
CUP	1	1	0	2
AML	0	1	0	1
GCT	1	0	0	1
penile SCC	1	0	0	1
	GENIE	cbioportal	Baylor	Total
Database size	143425	65853	4160	213438

Supplemental Table 1. FGFR3-TACC3 fusion-positive cancers reported across a series of public databases. The first (red) column contains data from AACR GENIE cohort v12.0[1]. The second (blue) column contains data from a cbioportal set of non-redundant studies which included MSK-IMPACT, TCGA PanCancer Atlas, UMich Metastatic Solid Cancers, Broad/Dana-Farber MSS Mixed Solid Tumors, China Pan-cancer[2–8]. The third (green) column contains internal unpublished data from Baylor College of Medicine. Combined, the three databases contained genetic sequencing from 213,438 unique patients (gray column), of whom 168 had tumors containing FGFR3-TACC3 fusions, for an overall frequency of 0.079%.

Suppl. Table 2

	Age (years) @ collection of liquid biopsy	Most recent treatment (other than ADT)	TP53 p.G245D reads /total reads (%)	BRCA1 p.N1521fs reads /total reads (%)	FGFR3ex18-TACC3ex12 fusion reads /total reads (at FGFR3ex18)	BRAF p.G469A reads /total reads (%)	FGFR2 p.F276C reads/ total reads (%)	NRAS p.Q61L reads /total reads (%)
Liquid biopsy NGS	72.42	-	9/3767 (0.24%)	0/3549 (0.0%)	0/3508	0/2961 (0.0%)	1/3492 (0.03%)	2/2514 (0.08%)
	73.13	-	2/5137 (0.04%)	2/5023 (0.04%)	0/5014	0/4418 (0.0%)	0/4609 (0.0%)	0/3682 (0.0%)
	73.45	-	7/5707 (0.12%)	18/6235 (0.29%)	0/6159	0/5505 (0.0%)	0/5772 (0.0%)	2/4371 (0.05%)
	73.61	-	19/7723 (0.25%)	33/7571 (0.44%)	0/7252	0/7066 (0.0%)	5/7804 (0.06%)	5/5542 (0.09%)
	73.78	-	264/4718 (5.6%)	346/4796 (7.21%)	43/4524	0/4253 (0.0%)	0/4646 (0.0%)	2/3483 (0.06%)
	73.86	-	983/3625 (27.12%)	1323/4015 (32.95%)	215/4618	2/4508 (0.04%)	1/3769 (0.03%)	0/3332 (0.0%)
	73.89	-	2044/4762 (42.92%)	2539/5362 (47.35%)	429/6089	0/7030 (0.0%)	1/5141 (0.02%)	1/5092 (0.02%)
	73.97	Gemcitabine/Cisplatin	11/4968 (0.22%)	1/5307 (0.02%)	0/4905	0/4611 (0.0%)	1/5024 (0.02%)	0/3627 (0.0%)
	74.03	Gemcitabine/Cisplatin	11/5276 (0.21%)	0/5876 (0.0%)	0/5751	0/5241 (0.0%)	1/5545 (0.02%)	0/4319 (0.0%)
	74.09	Gemcitabine/Cisplatin	2/6659 (0.03%)	1/7302 (0.01%)	0/6722	0/6905 (0.0%)	3/6439 (0.05%)	2/5157 (0.04%)
	74.18	Pembrolizumab	5/8163 (0.06%)	0/8364 (0.0%)	0/8237	0/7526 (0.0%)	2/8329 (0.02%)	1/6262 (0.02%)
	74.35	Pembrolizumab	5/6267 (0.08%)	4/6289 (0.06%)	1/6290	0/5648 (0.0%)	2/5789 (0.03%)	1/4359 (0.02%)
	74.43	Pembrolizumab	16/6169 (0.26%)	33/6764 (0.49%)	2/4513	1/5870 (0.02%)	0/6216 (0.0%)	3/4667 (0.06%)
	74.53	Pembrolizumab	70/4340 (1.61%)	117/4537 (2.58%)	24/4421	0/3888 (0.0%)	2/4665 (0.04%)	0/3298 (0.0%)
	74.58	Pembrolizumab	78/4234 (1.84%)	112/4546 (2.46%)	18/4505	0/4040 (0.0%)	0/4541 (0.0%)	1/3329 (0.03%)
	74.66	Pembrolizumab	349/4816 (7.25%)	461/5177 (8.9%)	88/5254	0/4976 (0.0%)	3/4832 (0.06%)	2/3790 (0.05%)
	74.83	Pembrolizumab	1160/6189 (18.74%)	1316/6293 (20.91%)	590/7921	0/7170 (0.0%)	40/5962 (0.67%)	2/5300 (0.04%)
	75.02	Pembrolizumab	2528/5145 (49.14%)	3298/6032 (54.68%)	1238/8890	2/8263 (0.02%)	44/5467 (0.8%)	14/5624 (0.25%)
	75.06	Radiation to spine	1301/4833 (26.92%)	2044/6019 (33.96%)	583/6561	0/7281 (0.0%)	114/5182 (2.2%)	7/5349 (0.13%)
	75.10	Radiation to spine	1743/8483 (20.55%)	2342/9458 (24.76%)	1003/11468	3/10146 (0.03%)	98/9226 (1.06%)	11/7973 (0.14%)
	75.14	Erdafitinib	149/6783 (2.2%)	295/7332 (4.02%)	49/7660	0/6521 (0.0%)	3/7642 (0.04%)	17/5224 (0.33%)
	75.16	Erdafitinib	282/7442 (3.79%)	530/7680 (6.9%)	1/7749	0/6914 (0.0%)	1/7814 (0.01%)	77/5352 (1.44%)
Tissue NGS	Primary ureteral tumor	-	238/258 (92.25%)	226/256 (88.28%)	0/375	150/639 (23.47%)	0/422 (0.0%)	0/544 (0.0%)
	Liver met	-	482/650 (74.15%)	391/570 (68.6%)	65/1540	0/984 (0.0%)	0/771 (0.0%)	0/1006 (0.0%)

Supplemental Table 2. Results of our post-hoc re-alignment and re-analysis of the raw FastQ files as described in the Methods section. Liquid biopsies are sorted on the top part of the table by timepoint of blood collection (patient age), while the two available UC tissue NGS samples are at the bottom. VAFs are provided for all point mutations (missense and indels), while for the FGFR3ex18-TACC3ex12 fusion it is provided as a ratio of fusion reads over total reads at FGFR3ex18 (which we use as a reference point).

Suppl. Table 3

	Age (years) @ collection of liquid biopsy	Most recent treatment (other than ADT)	[FGFR3ex18-TACC3ex12 reads /total FGFR3ex18 reads]/TP53 p.G245D VAF
Liquid biopsy NGS	72.42	-	0.000
	73.13	-	0.000
	73.45	-	0.000
	73.61	-	0.000
	73.78	-	0.170
	73.86	-	0.172
	73.89	-	0.164
	73.97	Gemcitabine/Cisplatin	0.000
	74.03	Gemcitabine/Cisplatin	0.000
	74.09	Gemcitabine/Cisplatin	0.000
	74.18	Pembrolizumab	0.000
	74.35	Pembrolizumab	0.199
	74.43	Pembrolizumab	0.171
	74.53	Pembrolizumab	0.337
	74.58	Pembrolizumab	0.217
	74.66	Pembrolizumab	0.231
	74.83	Pembrolizumab	0.397
	75.02	Pembrolizumab	0.283
	75.06	Radiation to spine	0.330
	75.10	Radiation to spine	0.426
	75.14	Erdafitinib	0.291
	75.16	Erdafitinib	0.003
Tissue NGS	Primary ureteral tumor	-	0.000
	Liver met	-	0.057

Supplemental Table 3. This table tracks the ratio of the FGFR3ex18-TACC3ex12 fusion frequency (defined as fusion reads/total reads at FGFR3ex18) to the TP53 p.G245D VAF in ctDNA over time, to demonstrate the relative frequency of the fusion-containing subclone as part of the total UC tumor burden through different phases of the clinical course. We selected the TP53 p.G245D VAF as a reference, because this variant appears to be the earliest known molecular finding in our patient's UC. The fusion-containing subclone appears to be more common at the time of clinical relapse (peak ratio 0.397) compared to at the time of initial UC diagnosis (peak ratio 0.172). This ratio then falls rapidly after initiation of erdafitinib (nadir ratio 0.003), representing the rapid elimination of the FGFR3ex18-TACC3ex12 fusion subclone due to sensitivity to erdafitinib. For comparison, this ratio was 0.057 in the liver metastasis when biopsied at the time of initial UC diagnosis.

REFERENCES

1. AACR Project GENIE: Powering Precision Medicine through an International Consortium. *Cancer Discov.* **7**, 818–831 (2017).
2. Cerami1, E. *et al.* The cBio Cancer Genomics Portal: An Open Platform for Exploring Multidimensional Cancer Genomics Data. *Cancer Discov.* **2**, 401–404 (2012).
3. Gao, J. *et al.* Integrative Analysis of Complex Cancer Genomics and Clinical Profiles Using the cBioPortal. *Sci. Signal.* **6**, pl1 (2013).
4. Zehir, A. *et al.* Mutational Landscape of Metastatic Cancer Revealed from Prospective Clinical Sequencing of 10,000 Patients. *Nat. Med.* **23**, 703–713 (2017).
5. Robinson, D. R. *et al.* Integrative Clinical Genomics of Metastatic Cancer. *Nature* **548**, 297–303 (2017).
6. Miao, D. *et al.* Genomic correlates of response to immune checkpoint blockade in microsatellite-stable solid tumors. *Nat. Genet.* **50**, 1271–1281 (2018).
7. Wu, L. *et al.* Landscape of somatic alterations in large-scale solid tumors from an Asian population. *Nat. Commun.* **13**, 4264 (2022).
8. Aaltonen, L. A. *et al.* Pan-cancer analysis of whole genomes. *Nature* **578**, 82–93 (2020).
